# H3K18Ac as a Marker of Cancer Progression and Potential Target of Anti-Cancer Therapy

**DOI:** 10.3390/cells8050485

**Published:** 2019-05-22

**Authors:** Marta Hałasa, Anna Wawruszak, Alicja Przybyszewska, Anna Jaruga, Małgorzata Guz, Joanna Kałafut, Andrzej Stepulak, Marek Cybulski

**Affiliations:** 1 Department of Biochemistry and Molecular Biology, Medical University of Lublin, Chodzki 1 St., 20-093 Lublin, Poland; anna.wawruszak@umlub.pl (A.W.); alicja.przybyszewska@umlub.pl (A.P.); anna.jaruga@umlub.pl (A.J.); malgorzata.guz@umlub.pl (M.G.); joanna.kalafut@umlub.pl (J.K.); andrzej.stepulak@umlub.pl (A.S.); marek.cybulski@umlub.pl (M.C.); 2 Postgraduate School of Molecular Medicine, Medical University of Warsaw, Trojdena 2a St., 02-091 Warsaw, Poland; anna.jaruga@umlub.pl

**Keywords:** histone acetylation, histone deacetylation, HATs, HDACs, H3K18Ac, cancer hallmarks

## Abstract

Acetylation and deacetylation are posttranslational modifications (PTMs) which affect the regulation of chromatin structure and its remodeling. Acetylation of histone 3 at lysine placed on position 18 (H3K18Ac) plays an important role in driving progression of many types of cancer, including breast, colon, lung, hepatocellular, pancreatic, prostate, and thyroid cancer. The aim of this review is to analyze and discuss the newest findings regarding the role of H3K18Ac and acetylation of other histones in carcinogenesis. We summarize the level of H3K18Ac in different cancer cell lines and analyze its association with patients’ outcomes, including overall survival (OS), progression-free survival (PFS), and disease-free survival (DFS). Finally, we describe future perspectives of cancer therapeutic strategies based on H3K18 modifications.

## 1. Introduction

In eukaryotic cells, chromatin structure is highly organized and is divided into basic repeating units called nucleosomes. The one-fold nucleosome is constructed from a positively-charged histone octamer (which encompasses two copies of each H2A, H2B, H3, and H4) and 147 bp of negatively-charged DNA that encircles the octamer 1.7 times [1]. Histone H1 is a 10–80 bp DNA linker which separates adjacent nucleosomes form each other. Interestingly, H1 is strongly associated with repression of gene expression and promotion of DNA methylation. Moreover, the C-terminal domain of H1 interacts with DNA methyltransferases such as DNA (cytosine-5)-methyltransferase 1 (DMNT_1_) and DNA (cytosine-5-)-methyltransferase 3 beta (DMNT3b), which is connected with carcinogenesis [2,3,4,5]. 

**Figure 1:**
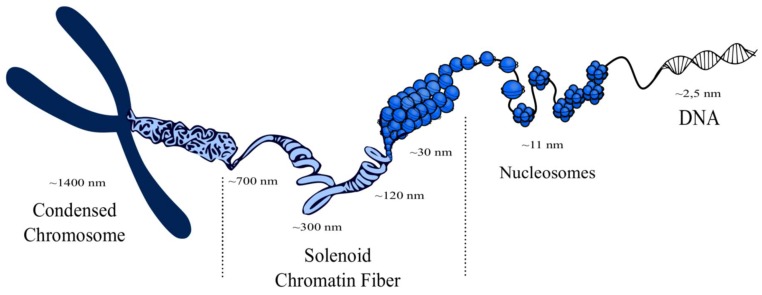
Visualization of a chromosome structure. Histone octamers with DNA are compressed in nucleosomes, which in turn form chromatin fibers called solenoids. Solenoids form the structure of a chromosome, which is located in the nucleus [10,11,12,13].

The nucleosomes form an intermediate level of chromatin structure consisting of chromatin fibers, called solenoids, which have a diameter of 30 nm, 120 nm, 300 nm, and 700 nm and which finally are arranged in a mitotic chromosome [6,7]. Interestingly, it has been found that nucleosomes form separate groups called clutches, which have different size and density and are interspersed via nucleosome-exhausted regions. Moreover, a strong correlation exists between spatial orientation, size, and spatial density of nucleosomes and pluripotency of cells. Steam cells mark out clutches with low density of nucleosomes. Furthermore, bigger-sized clutches with higher spatial density of nucleosomes and higher content of H1 are associated with heterochromatin, in contrast to smaller clusters with lower spatial density of nucleosomes, which are associated with euchromatin [8]. Chromatin fibers are highly organized and form chromosomes in the nucleus. The chromatin structure from a single histone octamer to condensed chromosome is presented below (Figure 1) [9]. 

N-tails of histones within the nucleosome octamer are considered a well-known target for specific chromatin epigenetic posttranslational modifications (PTMs) [14,15]. PTMs are associated with synthesis and subcellular localization of proteins as well as with their enzymatic activity. They recognize internal and external stimulations and they are able to respond through signal transmission and its amplification. PTMs are also able to regulate cellular metabolism and pathways of signal transduction [16]. 

In opposition to DNA mutations, PTMs highly affect nucleic acids and proteins, which is crucial for chromatin structure regulation without DNA sequence changing. These chromatin modifications have an influence on DNA transcriptional machinery, which in turns affects gene expression. PTMs are involved in carcinogenesis, as they are able to silence tumor-suppressor genes as well as enhance expression of oncogenes [17].

Epigenetic alterations in chromatin structure could occur as a direct result of modifier’s operations through chromatin-remodeling complexes such as SWItch/Sucrose Non-Fermentable SWI/SNF [18] and non-coding RNAs [19], as well as indirectly through modification of chromatin binding molecules [20]. Chromatin-structured modifications are connected with various types of covalent reactions, including acetylation, methylation, phosphorylation, and ubiquitylation on histones [21,22,23,24,25].

The most common epigenetic histone modifications are acetylation, which is described in this chapter, and methylation. Reversible histone methylation and demethylation occur at slower rates than many other PTMs. These modifications are catalyzed by methyltransferases (KMTs) and demethylases (KDMs), which add and remove methyl groups, respectively. Interestingly, lysine can be mono-, di-, and tri-methylated on its ε-amine group, and it regulates different cellular processes, such as translation and transcription [19]. Most KMTs seem to be highly selective with respect to specific lysine residue and the methylation degree of this lysine, and they are able to methylate non-histone substrates as well [26]. The investigation of alterations in the methylation network present in cancer cells provides new perspectives in cancer therapy by inventing small molecule inhibitors of KMTs and KDMs [27]. 

**Figure 2:**
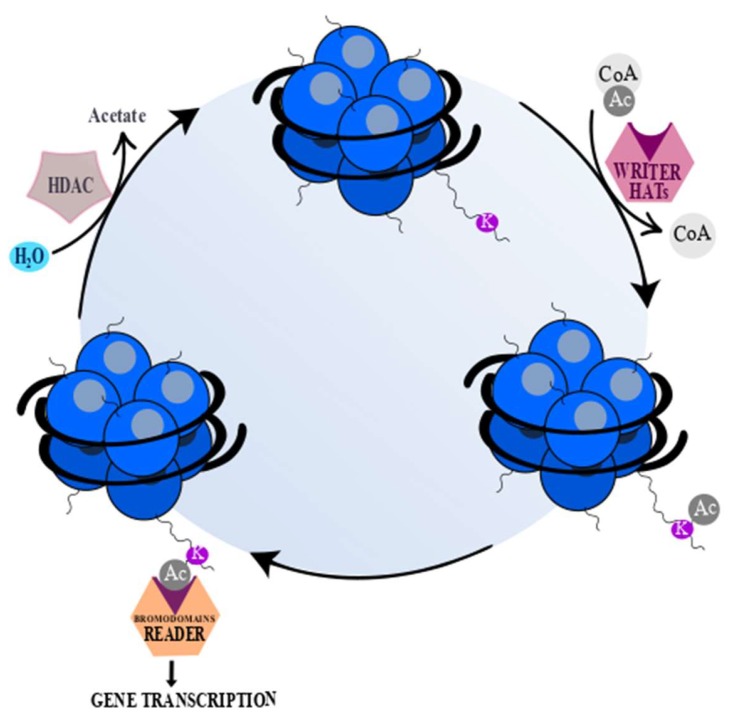
“Writers”, “readers”, and “erasers”. “Writers” (histone acetyltransferases (HATs)) (pink) catalyze the transfer of an acetyl group to a histone lysine (K) or arginine residue. “Erasers” (histone deacetylase proteins (HDACs)) (gray) catalyze removal of an acetyl group from acetylated lysine or arginine. “Readers” (orange) possess specialized domains which are able to recognize and interact with certain histone modifications [31,32]. The acetyl (Ac) groups are added by HATs and removed by HDACs. Acetyl coenzyme A (Acetyl-CoA) serves as a donor of Ac groups and it is mainly obtained through ATP-citrate lyase reaction, under which mitochondrial-derived citrate is converted to acetyl-CoA and oxaloacetate. The changes of ATP-citrate lyase activity lead to variable acetyl-CoA accessibility and as a consequence global histone acetylation [20]. The deacetylation by HDACs yields deacetylated peptide and free acetate [20,33]. However, sirtuins (Class III HDACs), except for deacetylated peptide, generate a mixture of 2′- and 3′-O-acetyl-ADP ribose and nicotinamide [33].

Epigenetic modifications are highly dependent on three groups of proteins. The first group, called “writers”, is able to transfer specific groups, depending on the type of reaction, to DNA or lysine/arginine residues on histones, such as histone acetyltransferases (HATs), during acetylation [28,29]. The next group, “readers”, possesses highly-conserved bromodomains (BrDs) that contain specific and conserved residues responsible for recognition of acetyl-lysine. Acetylated lysine is surrounded by other residues placed in bromodomain and, ipso facto, these residues contribute to binding specificity. Moreover, a lot of bromodomains are able to bind to more than one acetyl-lysine residue simultaneously [29], which is needed to arrange a functional connection between acetylation of lysine residues and protein-protein interactions mediated by acetylation during gene transcription [30]. Bromodomains play a crucial role in signal transduction from acetylated lysine residues and subsequently in the translation into normal as well as abnormal cell phenotypes [28,30]. The third group of chromatin-modifying proteins includes histone deacetylase proteins (HDACs), called epigenetic “erasers”, which are enzymes that are able to remove the acetyl-group from an ε-N-acetyl-lysine amino acid localized in the N-terminus of histones [28,29]. The mechanism of epigenetic modifications is presented below (Figure 2).

**Figure 3:**
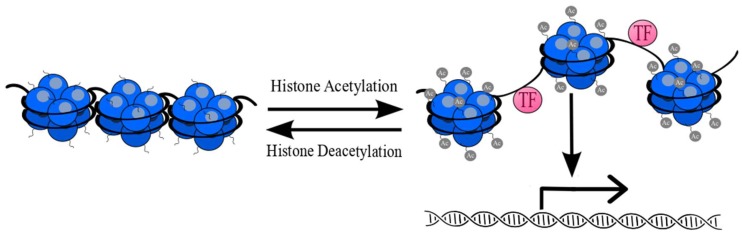
Reversible posttranslational acetylation of histones (blue). Acetylation of lysine residues causes relaxation of chromatin structure and provides easier access for transcription factors (TF) (pink). Removal of acetyl-groups (gray) from lysine residues leads to chromatin compaction and its inactivation [38].

The balance between acetylation and deacetylation is crucial for chromatin stability and integrity [33,34,35,36]. Removal of acetyl-groups from lysine residues by HDACs has an impact on chromatin compaction through interaction between positively charged N-tail histone residues and negatively charged DNA. Chromatin repression by HDACs inactivates transcription through restraining access for transcriptional factors at gene promoter regions [37]. An overview of the balance between acetylation and deacetylation is presented in Figure 3.

Basically, HDACs have been considered as being transcriptional repressors, but there is evidence that HDACs can also activate gene transcription [39,40,41,42,43]. Genes may be expressed because of a DAC-dependent deactivation of a gene coding for a transcriptional repressor. Next to histone deacetylation, HDACs are able to deacetylate non-histone proteins including transcription factors such as tumor suppressor p53 [44] and nuclear factor kappa-light-chain-enhancer of activated B cells NF-κB [45,46], as well as signal mediators such as signal transducer and activator of transcription 3 STAT3 [47], and Smad7 oncoprotein [48].

In opposition to HDACs, residues of p53 acetylated by HATs may be located in variable sites, which leads to elevation of p53 DNA binding or loss of its transcriptional activity. It has been demonstrated that mutation of the C-terminal site of p53, where acetylation occurs, prompts comprehensively the loss of p53-dependent cyclin-dependent kinase inhibitor p21 transcription [49,50]. Acetylation of signal mediators may be prominent in subsequent stages in cancer progression. For example, STAT3 acetylation has an impact on DNA methylation by methyltransferase 1 (DNMT_1_), and as a consequence induces silencing of tumor-suppressor genes [51]. Taken together, the acetylation of non-histone targets is crucial for their activation or inactivation. The non-histone targets, which are part of multi-molecular complexes, are associated with a wide spectrum of cellular activities, including cell proliferation, survival, and apoptosis, as well as chromatin remodeling [52]. In general, histone acetylation occurs in two different states. Hyperacetylation, which is the first of these states, is properly applied when all or most of N-terminal lysines located on histones are in acetylated form. In contrast, hypoacetylation often includes non-acetylated or single-acetylated N-terminal lysines [53]. Reversible acetylation of histones is strongly associated with various types of cancer of different origin, including blood [54], bladder [55], brain [56], breast [57], colon [58,59,60], esophageal [61], gastric [62,63], liver [64], lung [65], pancreatic [66], prostate [67], and oral cancer [68].

Widespread research has been carried out on the acetylation level of lysine residues on histones 3 (H3) and 4 (H4). It has been demonstrated that the most important positions for acetylation are lysines placed at positions 9, 14, 18, and 27 on the N-tail of H3 (Lys9, Lys14, Lys18, and Lys27 respectively), and lysines placed at positions 5, 8, 12, and 16 on the N-tail of H4 (Lys5, Lys8, Lys12, and Lys16, respectively) [36].

## 2. Histone Acetyltransferases and Histone Deacetylases

This chapter presents a short description and classification of HATs and HDACs and the usefulness of HDAC inhibitors in cancer therapy [69]. 

### 2.1. HAT Classification

Histone acetyltransferases are able to position acetyl groups into lysine residues during lysine acetylation [70]. They are able to acetylate histone proteins as well as non-histone proteins. HATs are divided into two groups according to their localization in a cell as well as their substrate specificities. The first group, called A HATs, exclusively occurs in the nucleosome, and therefore has an influence on chromatin remodeling by modification of histones connected with chromatin. The second group, called B HATs, is present in the nucleosome and cytoplasm simultaneously, and is able to acetylate free soluble histones [71]. The subfamilies of HATs have been classified according to their sequence homology and their functional roles [72]. The largest subfamily of acetyltransferases (the MYST family) consists of MOF, MOZ, MORF, HBO1, and TIP60. They possess a highly conserved MYST-domain, which consists of a zinc finger and acetyl-CoA-binding motif. Some of them share additional structural figures, including plant homeodomain-linked zinc fingers (MORF and MOZ) and chromodomain (TIP60 and MOF) [73,74]. They occur as part of bigger, multi-subunit protein complexes, which are involved in pro- and anti-oncogenic activities [73]. The next group is the Gcn5-related N-acetyltransferases (GNATs) family, represented by p300/CREB binding protein (CBP) associating factor (PCAF), HAT1, and GCN5, which possess bromodomains and are responsible for lysine acetylation of histones H2B, H3, and H4 [72,75]. The CBP/p300 subfamily has a lot of small domains which interact with many other proteins with disordered transactivation domains, including NF-κB and p53 [76]. 

### 2.2. HDAC Classification and Their Inhibitors

According to the homology for yeast HDACs, 18 human HDACs have been identified and have been classified into four classes [77]. Class I HDACs share homology with transcriptional regulator Rpd3 in yeast and are localized in the nucleus. Class I HDACs encompasses HDACs 1, 2, 3, and 8 [78]. Catalytic domains of HDAC1 and HDAC2 are almost identical, in addition to their C-terminal tails, which contain tandem casein kinase 2 (CK2) phosphorylation sites [79]. Moreover, HDAC1 and HDAC2 occur as part of larger, multiprotein complexes, which are able to mediate acetyl group transfer, including Sin3, Mi-2/NuRD, CoREST, and NCoR/SMRT complexes [80,81]. Class II HDACs are closely related to Hda1 and may be subdivided into class IIa (HDAC4, 5, 7, and 9) and class IIb (HDAC6 and 10). Class II HDACs may shuttle between the cytoplasm and nucleus. Hence, class II HDACs are able to deacetylate non-histone proteins in cytoplasm [78]. Class III HDACs (sirtuins) include SIRT1, 2, 3, 4, 5, 6, and 7, which are homologous with Sir2 in yeast and are required for activation of the coenzyme NAD^+^ [82]. Class IV HDACs (HDAC 11) are homologous with class I and II enzymes and are Zn^2+^ dependent [78].

Aberrant expression of HDACs have been noticed in many human diseases, including cancer, making them important therapeutic targets [83]. Histone deacetylase inhibitors (HDIs) are a new class of chemotherapeutic agents which block activity of HDACs. HDIs alter gene expression by enhancing the acetylation status of histones and in consequence the induction of chromatin relaxation [84]. Many structurally diverse HDIs isolated from natural sources or synthetically produced compounds have been identified [85]. 

HDIs have been classified into five basic groups: hydroxamic acids (vorinostat, belinostat, panobinostat, givinostat, resminostat, abexinostat, quisinostat, rocilinostat, and practinostat), short chain fatty acids (valproic acid, butyric acid, and phenylbutyric acid), benzamides (entinostat, mocetinostat, tacedinaline, and 4SC202), cyclic tetrapeptides (romidepsin), and sirtuin inhibitors (nicotinamide, cambinol, sirtinol, and EX-527) [69]. Many different HDIs have been invented which inhibit activity of most HDACs. Some HDIs are pan-inhibitors which inhibit activity of several different HDACs (phenylbutyrate, vorinostat, and belinostat), while some selectively inhibit only one or two HDACs (for example, valproic acid and sodium butyrate inhibit both HDAC I and IIa). Bezamides, including entinostat, mocetinostat, and domatinostat, as well as cyclic peptides, including romidepsin and apicidin, exclusively inhibit HDAC I only [86]. It has been shown that HDIs induce apoptosis, differentiation, and growth arrest, and inhibit angiogenesis, in many types of cancers [85]. Interestingly, it has been noted that pan-HDAC inhibitors, e.g., AR42 and sodium valproate, are able to alter the immunogenicity of melanoma cells [87].

A special class of HDIs are the sirtuin inhibitors, which are divided into two groups. The first group– consists of agents which interact with the NAD^+^ binding site, and the second consists of molecules interacting with the acetyl lysine binding site. Nicotinamide is widely used as an inhibitor of sirtuins, as inhibition of sirtuin activity in cells has been reported after treatment with high concentrations of NAD^+^ [88]. Most sirtuin inhibitors inactivate SIRT1, 2, and 3. These inhibitors are AGK2, cambinol, salermide, sirtinol, splitomicin, suramin, and tenovin. Splitomicin weakly inhibits SIRT1, while sirtinol inhibits only SIRT2. Cambinol, AGK2, tenovin-6, and salermide inhibit both SIRT1 and SIRT2 [89]. Interestingly, IC_50_ value for AGK2 and tenovin-6 are similar for both SIRT1 and SIRT2 [90], while suramin inhibits SIRT1, SIRT2, and SIRT5 (IC_50_ 0.297 µM, 1.15 µM and 22 µM, respectively) [89].

A large number of studies have revealed tumor specific and more efficient anticancer activities of HDIs used in combination with other drugs [84]. It has been demonstrated that HDIs are relatively non-toxic to normal cells but show selective anti-proliferative activity against many types of cancer cells, despite histone hyperacetylation being induced by HDIs in both cell types. The tumor selectivity and ability of HDIs to induce apoptosis may result from the regulation of the G2 checkpoint within the cell cycle. In normal cells the G2 checkpoint is activated by HDIs, which causes inhibition of the cell cycle, but this is dysregulated in tumor cells [85].

## 3. Histone 3 Modifications as Biomarkers of Cancer Progression

It has been demonstrated that HATs play a dual role in carcinogenesis. They can act like a tumor suppressor through inhibition of cancer cells proliferation or as oncogenes by increasing activity of pro-oncogenic proteins via deregulated acetylation. The role of HATs in carcinogenesis depends on the site of acetylation of target proteins and the type of cancer [91]. Importantly, histone hypoacetylation is considered a biomarker of prognosis in many types of cancers [92].

It has been demonstrated that global increase of acetyl-lysine placed in position 4 on the N-tail of histone 3 (H3K4Ac) in breast cancer cell lines (MCF7 and MDA-MB-231) and global rising of H3K4Me3 in MDA-MB-231 cell lines are associated with cancer cell metastasis. H3K4Ac is considered a predictor for both initial transformation and aggressive metastatic phenotypes of breast cancer [93]. Moreover, it has been noted that acetylation of H3K4 is strongly connected with TIP60 expression in breast cancer. The influence of under-expression of TIP60 in breast cancer has been tested using athymic Balb-c mice, in which the TIP60 knockdown was induced in MDA-MB-231 (ER+) and MCF-7 (ER-) cell lines. The experiment showed that TIP60 is able to affect reduction of acetylation on H3K4 and that tumor development is increased in sh-TIP60 MDA-MB-231 xenografts, in contrast to sh-TIP60 MCF-7 xenografts, in which tumor development is inhibited. These findings suggest that expression of TIP60 occurs in different subtypes of breast cancer and that it changes the level of histone acetylation. Additionally, both TIP60 and H3K4 are placed in the same promoter region of genes connected with breast cancer. Inhibition of TIP60 may modify expression of genes dependent on steroid hormone regulation, which in turn is associated with promotion or inhibition of tumor development [8]. 

It has been found that the oncogenic N-Ras signaling pathway increases H3 acetylation (H3K9Ac and H3K23Ac) in embryonic kidney 293T cells [94]. It has been demonstrated that H3K14Ac is highly related to the tumor suppressor polybromo1 (PBRM1) in kidney cancer. PBRM1 contains six bromodomains (BDs) which are able to recognize and bind many acetylated lysines at histone 3. Interestingly, one BD, BD2, exclusively interacts with H3K14Ac rather than with other histone lysines. BD2 possesses a highly conserved asparagine at position 263 in the aminoacid sequence (Asn263), which seems to be crucial for acetyl-lysine recognition through hydrogen bond formation to acetyl groups [95,96].

It has been found that acetylation of H3K27 is associated with colon cancer associated transcript-1 (CCAT1) expression in esophageal squamous cell carcinoma (ESCC). High CCAT1 expression in ESCC has been found to correlate with the level of H3K27Ac, which is enriched at the CCAT1 promoter. The level of H3K27Ac is increased in cancer tissues compared to normal tissues, as well as in Eca-109 cells (ESCC cells) compared to normal human esophageal epithelial cells (HET-1A). Additionally, knockout of CCAT1 has been found to inhibit cell proliferation and migration both in vitro and in vivo [97]. H3K27Ac also upregulates and activates the placenta-specific protein 2 (PLAC2) gene in oral squamous cell carcinoma (OSCC). Expression of PLAC2 is enriched in OSCC cells (CAL-27 and SCC-9) compared with parental normal oral epithelial keratinocytes (HOK), and PLAC2 promotes progression of OSCC through regulation of the Wnt/β-catenin pathway [98]. H3K27Ac is enriched at the promoter region of terminal differentiation-induced non-coding RNA (TINCR), which promotes resistance to trastuzumab and epithelial-mesenchymal transition (EMT) in breast cancer. TINCR upregulation is mediated by the CBP enzyme which enhances H3K27 acetylation at the promoter region of TINCR [99]. Regulation of H3K27 acetylation and its methylation by HOX transcript antisense intergenic RNA (HOTAIR) leads to transcriptional activation or repression of E-cadherin that can induce the development of gastric cancer (GC) [100]. 

Another study using glioma U87EGFRvIII cells showed that the level of H3K56Ac is increased by rapamycin complex 2 (mTORC2), and that mTORC2 regulates glycolytic genes by influencing H3K56Ac levels at the promoters of these genes. Additionally, mTOR depletion leads to enhanced recruitment of SIRT6 to promoters of glycolytic genes, which in turn allows metabolic reprogramming in glioma cells [101].

Except for acetylation of lysines on histone 3, as mentioned above, there are important lysine acetylations that occur on histone 4 which may also be considered as biomarkers of cancer progression. It has been discovered that activation of microglia by glioma cells is connected with global H4K16 acetylation and that a high level of H4K16Ac expression promotes a tumor-supporting phenotype in microglia. In contrast, H4K16Ac downregulation is associated with reduction of tumor-supporting behavior [102]. The latest data has shown that H4K16Ac takes part in RGP78 upregulation and AKT/4EBP1 activation, which is related to cell death. Surprisingly, docetaxel-resistant prostate cancer PC3/Doc cells are hyper-acetylated in comparison to parental PC3 cells, despite the fact of the higher level of HDAC5 in resistant prostate cells. These findings suggest that HK16Ac may be used as a marker for effectiveness of HDI treatment in prostate cancer. Moreover, the results support the validity of HDI pre-clinical application in the treatment of metastatic castration-refractory prostate cancer (mCRPC) [103]. The most important data about the expression of histone markers in cancer cell lines are shown in Table 1.

**Figure 4:**
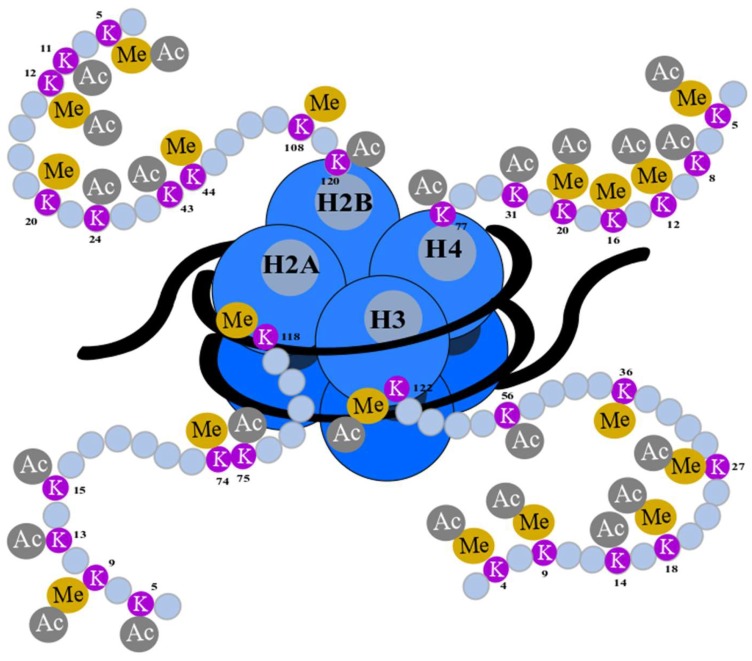
Epigenetic modifications of a histone N-terminus. Lysine (K) residues are located at the N-terminal (gray) of histones: H2A, H2B, H3, and H4 (blue) and encircled DNA (black). They undergo posttranslational modifications such as acetylation (Ac) and methylation (Me). The numbers of amino acids refer to their position in the sequence of histones [36,104,105].

Taken together, H3 and H4 possess a large number of lysines, which undergo epigenetic modifications, including acetylation and methylation. The knowledge of these modifications can be used in cancer prognosis and therapy. Particular lysine residues and their epigenetic modifications are presented in Figure 4.

## 4. H3K18Ac as a Biomarker in Cancer Progression

This chapter presents the results of studies from the last 10 years connected with H3K18 acetylation and its role in the progression of the most common human solid tumors. HDAC activity is discussed in the context of histone and non-histone protein acetylation. The relationship between acetylation and cancer progression is demonstrated at the epigenetic, cellular, and tissue level. Hypoacetylation of H3K18 is presented in respect to cancer clinical staging. 

### 4.1. Prostate Cancer

Microarray-based comparative analysis of HAT activity in the hormone-sensitive (HS) prostate cancer cell line (LNCaP) and its castrate-resistant (CR) daughter cell line (C4−2) has revealed increased HAT activity against specific histone sites of H3 in the CR cell line compared to its HS equivalent [106]. The progression of HS to CR is accompanied by histone H3 lysine 18 (H3K18) hyperacetylation, upregulation of p300 activity, and downregulation of SIRT2 protein expression. These findings suggest that enhanced HAT activity in C4-2 cells can be assigned to activated, acetylated p300, and that SIRT2 regulates the acetylation level of the activated acetyl-p300 form [106]. The expression of histone H3K18Ac acetylation, and proteins that regulate its acetylation (P300) and deacetylation (SIRT2), has been evaluated in benign prostatic hyperplasia (BPH), high grade prostatic intraepithelial neoplasia (HGPIN), prostate cancer (PCa), and metastases [107]. The levels of H3K18Ac were found to be higher in primary cancers and metastases compared to benign tissues and increased H3K18Ac identified patients at increased risk of PCa recurrence. Moreover, downregulation of P300 protein expression in PCa and metastases and a progressive loss of SIRT2 compared to benign, malignant, and metastatic tissues has been observed. Analysis of genomic and clinical data in The Cancer Genome Atlas (TCGA) and Gene Expression Omnibus (GEO) has revealed that the HAT P300 and its target H3K18Ac increase during prostate cancer progression, while the HDAC SIRT2 decreases. Gain of H3K18Ac and loss of SIRT2 reflect P300 mediated hyperacetylation, and their determination may help identify patients likely to benefit from therapy with HAT inhibitors [107]. It has been demonstrated that SIRT7 plays a vital role in the aggressiveness of prostate cancer, meaning it is a promising predictive marker for aggressive prostate cancer [108]. SIRT7 levels have been found to be elevated in tumors and positively associated with the tumor grade. The knock down of SIRT7 inhibits the migration of two androgen-independent prostate cancer cells (DU145 and PC3), although the overexpression of the native protein, but not the mutated form, promotes cell migration and invasion of the poorly aggressive, androgen-dependent LNCaP cell line. SIRT7 overexpression induces resistance of cells to docetaxel, which indicates that SIRT7 deacetylase activity is associated with resistance to chemotherapy [108].

### 4.2. Pancreatic Cancer

The influence of acetylation and deacetylation, which regulates the expression of oncogenes and tumor suppressor genes, on pancreatic carcinogenesis is not well known. It has been shown [66]. that low expression of H3K18 acetylation in patients with stage I and II pancreatic adenocarcinomas is an independent predictor of poor survival. In contrast to other research [109], high H3K18Ac expression in pancreatic cancer has been found to be an independent prognostic factor for poorer survival, and H3K18Ac expression is lower in nonmalignant tissues compared to the primary tumors and metastases [109].

### 4.3. Colon Cancer

Expression of H4K12Ac and HDAC2, but not H3K18Ac, has been found to rise from normal tissue through adenoma to moderately and well-differentiated colorectal carcinoma (CRC), suggesting that HDAC2 and H4K12Ac together may play a role in the progression of colon cancer. Additionally, HDAC2 has the diagnostic power to differentiate between cancer and non-cancer diagnosis. The acetylation of H4K12 and H3K18 is decreased in poorly differentiated compared to moderately and well differentiated cancer cells [110]. Selective agents are being sought that might target abnormal patterns of histone modification as a means of destroying cancer cells. The differentiation status of cancer cells is important in regard to chromatin modification, and, hence, further studies will expand the current work using wide-genome array-based techniques to investigate histone modification and HDAC2 expression in colorectal adenoma and CRCs with different levels of differentiation. The results to date are encouraging because they demonstrate that aberrant expression of HDAC2 frequently occurs in patients with CRC, providing potential biomarkers for use in future clinical trials. Another study performed on colon cancer has demonstrated that GPR109A, the receptor for short-chain fatty acids, functions as a tumor inhibitor in CRC, and the IFNγ can be used to activate GPR109A transcription silenced by DNA methylation. The treatment of tumor cells with IFNγ removes the silencing of GPR109A without changing the methylation of its promoter, suggesting that histone acetylation may be critical in the IFNγ-induced expression of GPR109A. It has been discovered that IFNγ rapidly activates pSTAT1, which binds to the p300 promoter to activate its transcription, upon which p300 binds to the GPR109A promoter to induce H3K18 hyperacetylation, resulting in activation of GPR109 transcription. This study has shown that the IFNγ-producing cells of the host immune system counteract the silencing of GPR109A mediated by DNA methylation to suppress cancer development [111].

### 4.4. Breast Cancer

It is known that specific marks such as H3K18Ac are associated with transcriptionally active gene promoters. It has been discovered that most breast tumors score low for H4K16Ac, whereas H3K18ac and H4K20Me3 are expressed at relatively high levels. Low levels of H3K18Ac are associated with high tumor grade, and high expression of histone modifications is correlated with cancers positive for steroid receptors (androgen receptor, estrogen receptor, and progesterone receptor), increased expression of E-cadherin and BRCA1, and low p53 and HER-2 expressions. These findings may explain the poor prognosis of breast cancer patients with low levels of histone modifications and underline the biological importance of histone modifications. Indeed, decreased levels of histone modifications have been correlated with an unfavorable patient outcome, and the increased level of H3K18Ac detection has been associated with more advantageous breast cancer-specific survival (BCSS), longer disease-free survival (DFS), and metastatic-specific survival (MSS). Importantly, multivariate analysis has shown that the H3K18ac level is independent of other key prognostic factors including tumor size, histologic grade, and lymph node stage, with respect to BCSS and DFS. However, the correlations between histone modifications and patient outcome have been seen to be diminished in patients treated with hormonal therapy or chemotherapy. Nonetheless, this research supports the evidence that global hypoacetylation of H3K18 is demonstrative of cell transformation and may be a crucial prognostic marker in breast cancer [57].

### 4.5. Hepatocellular Carcinoma

It has been reported that SIRT7 has a high selectivity for acetylated H3K18 and that it supports malignant phenotype. It has been observed that HBx oncoprotein of hepatitis B virus (HBV) stabilizes SIRT7 and stimulates H3K18 deacetylation, and depletion of SIRT7 decreases cell viability and transformation. These findings show that SIRT7 is an important regulator of HBx-driven oncogenic transformation [112]. In other research, high expressions of SIRT7 and H3K18Ac in hepatocellular carcinoma (HCC) were associated with worse patient overall survival (OS), and H3K18Ac levels turned out to be an independent prognostic factor in multivariate analysis. SIRT7 expression and higher H3K18ac levels were observed in HCC cells compared to non-tumor hepatocytes, and SIRT7 expression was weakly correlated with H3K18Ac. These results suggest that other mechanisms may be involved in deacetylation of H3K18Ac in HCC [113]. Another study [114] has shown that up-regulation of the acetylation of histone 3 at the maternally expressed 3 (Meg3) differentially methylated region (DMR) increases the level of miR-376a that contributes to the development of HCC. Interestingly, HDAC9, a histone deacetylase responsible for H3K18 deacetylation, was established as the target of miR-376a, and its inhibition was found to increase the expression of miR-376a by up-regulating the global histone H3K18Ac. Finally, both miR-376a and HDAC9 were inversely correlated in HCC [114].

### 4.6. Lung Cancer 

It has been demonstrated [115] that overexpression of inhibitor of growth 5 (ING5) leads to p300 HAT activation and increased acetylation of p300 target proteins (p53 at K382 and H3 at K18) in the human NSCLC A549 cell line. C646, a specific p300 HAT inhibitor, has been found to reduce ING5-induced acetylation of p53K382 and H3K18 and subsequent expression of Bax and p21 proteins. These findings suggest that ING5 functions as a tumor suppressor by regulating expression of many genes through specific lysine acetylation and that it inhibits invasiveness of lung cancer cells [115].

### 4.7. Thyroid Cancer

It has been shown [116] that levels of H3K9-K14Ac are higher in thyroid follicular adenomas and carcinomas (papillary, follicular, and undifferentiated) than in normal tissues. Similarly, acetylated H3K18 levels have been found to increase in adenomas and cancers (except undifferentiated tumors) compared to normal tissues, indicating that reduction of H3K18Ac may play a role during thyroid cancer progression. The induction of RAS, BRAF, and RET–PTC oncogenes that are typically activated in thyroid cancers leads to an increase in either H3K9–K14ac or H3K18ac levels in rat thyroid cell lines. Moreover, thyroid stimulating hormone (TSH) increases levels of H3K18Ac in non-tumorigenic FRTL-5 rat thyroid cells, showing that hormonal stimulation and oncogene activation in the course of neoplastic transformation can modify levels of histone acetylation in thyroid cells [116].

## 5. Alteration of H3K18 Acetylation via Viral Oncoprotein: Adenovirus E1A 

It has been suggested that adenovirus E1A, a viral oncoprotein, influences global histone modifications via genome-wide redistribution of different histone-modifying enzymes such as p300/CBP. E1A has a strong influence on the global reduction of H3K18Ac (about 70%) compared to uninfected cells. Interestingly, in cells with late stage E1A infection, p300/CBP quantitatively interacts with E1A, bringing insight into the fact that direct interaction between p300/CBP and E1A can lead to hypoacetylation of H3K18Ac [117].

E1A is associated with 4000 primary and secondary targets because of its dynamic and flexible structure [118], and viral pre-mRNA is alternatively spliced into several transcripts. The 12S and 13S mRNAs, generated from two 5′ splice sites, and a common 3′ splice site, are most abundant in the early stage after infection and in transformed cells [119]. Each of these mRNAs encodes the large 289-amino acid E1A (E1A 289R) and the small 243-aminoacid e1a (E1A 243R) regulatory proteins, respectively, and both of them affect gene expression by interaction with other proteins through four conserved regions (CR), these being 1, 2, 3, and 4 [120]. CR1 and CR2 domains of both small and large E1A interact with histone HAT p300/CBP and the retinoblastoma (RB) family of proteins (RB1, p107, and p130) which reprogram gene expression in host cells and allow quiescent cells to enter the S-phase [121]. The transcriptional adaptor zinc finger-2 (TAZ2) domain on p300/CBP is a primary binding site of the E1A CR1 region [122]. Additionally, CR3, a highly conserved region of E1A proteins among different adenoviruses which is unique for E1A 289R, binds coactivators, including the aforementioned p300 and PCAF and repressors of transactivation, like GCN5 and BS69 [123]. Several studies have reported E1A’s ability to inhibit or activate histone acetyltransferase activity of p300/CBP [124]. CBP/p300 function as tumor suppressor proteins that interact with transcription factors and different regulatory proteins by acting as key molecular integrators which are crucial for cell proliferation, differentiation, apoptosis, and DNA repair [125]. Molecular abnormalities in expression of CBP and p300 are associated with gastrointestinal malignancies, acute myeloid leukemia and lung cancer [125,126,127]. Both CBP and p300 acetylate several histone lysines, including H3K18Ac. Chromatin immunoprecipitation combined with DNA microarrays studies (CHIP-chip) has confirmed that the interaction of small e1a oncoprotein with p300/CBP causes an around 70% reduction in cellular levels of H3K18Ac at promoters of most genes and increases H3K18Ac levels at promoters of genes that play key roles in the cell cycle or DNA replication [128]. It has been demonstrated [129] that the major repression function of E1A 243R oncoprotein resides within its N-terminal 1-80 amino acids domain. This domain is sufficient for inhibition of H3K18 acetylation and p300 mediated H3K18 acetylation in reconstituted chromatin in vivo, and it is known that hypoacetylation of histone H3 at this position is correlated with aggressive tumor phenotypes and poor prognosis in different cancers [129,130,131]. In cancer cells, the N-terminal domain of E1A represses MYC transcription through pathways involving targeting of both p300 and TRRAP, as well as inhibition of H3K18 and H4K16 acetylation, at the MYC promoter. The family of the MYC oncogene is deregulated in more than 50% of human cancers and its members participate in many aspects of oncogenesis, such as regulation of proliferation, apoptosis, differentiation, and metabolism of cells [132]. The repression of MYC expression via epigenetic modifications may become a novel anticancer therapy in the future.

## 6. The Role of H3K18Ac in Regulation of Cancer Hallmarks 

### 6.1. H3K18 Deacetylation by SIRT6 and SIRT7

Hypoacetylation of H3K18 is associated with aggressive tumor phenotypes and poor prognosis in patients with several cancer types [131]. It has been discovered that that SIRT6 actively removes acetyl groups from histones H3K9, H3K18, and H3K27, but that it has relatively low activity toward histones H3K4 and K3K23, and poor activity toward histones H3K14, H3K36, H3K56, and H3K79. The overexpression of SIRT6 observed in prostate cancer cells and downregulation of H3K18Ac suggests that SIRT6 influences prostate cancer development and that it can be a potential target for anti-cancer therapy [133]. 

It has been demonstrated that SIRT7 is essential for maintaining the cancer cell phenotype and stabilizing the tumorigenicity of tumors by selective deacetylation of H3K18Ac [134]. SIRT7 regulates fundamental cellular programs that participate in oncogenic transformation and tumor biology [135]. It controls expression of tumor-suppressive genes which stabilizes the transformed state of cancer cells via deacetylation of H3K18Ac at specific promoters [131,135]. SIRT7 also coordinates several molecular processes, inter alia tRNA and rRNA synthesis, which eventually promote the increased ribosome biogenesis vital for cancer cell growth, proliferation, and invasion. Inactivation of SIRT7 reverses the malignant phenotype of cancer cells and decreases their tumorigenicity [135]. It has been revealed that MYB binding protein 1, (Mybbp1), a component of the B-WICH chromatin remodeling complex, is capable of inhibiting the deacetylation of H3K18Ac by SIRT7, and it therefore may function as a tumor suppressor [134]. Deacetylation of H3K18Ac by SIRT7 is crucial for maintaining essential features of cancer cells, including escape from contact inhibition and anchorage-independent growth [131]. Additionally, SIRT7 is required for a global H3K18 hypoacetylation that leads to cellular transformation by the E1A viral oncoprotein. Importantly, depletion of SIRT7 markedly decreases the tumorigenicity of human cancer cell xenografts in vivo [131]. SIRT7 is enriched at promoters of different genes, including the tumor suppressor genes: nucleoside diphosphate kinase A (*NME1*), and COP9 signalosome complex subunit 2 (*COPS2*), as well as the ribosomal protein genes *RPS7*, *RPS14*, and *RPS20*. SIRT7 depletion induces acetylation of H3K18 at promoters of these genes, and as a consequence increases transcription of target genes [116].

### 6.2. H3K18Ac Deacetylation by HDAC1

The malignant transformation of human immortalized renal tubular epithelial HK-2 cells induced by chronic oxidative stress is accompanied by aberrant expression of genes involved in epigenetic modifications such as histone modifications (HDAC1, HMT1, and HAT1) and DNA methylation (DNMT1, DNMT3a, and MBD4) [136]. The decrease in global histone H3 acetylation including H3K18Ac is associated with up-regulation of HDAC1 expression [136], which is essential for mitosis in cancer cells and its deficiency-induced apoptosis mediated cell death [137]. Oxidative stress-induced hypoacetylation of histones has been reported at the global and gene-specific promoter level [138]. Transcriptional repression of p21 induced by H_2_O_2_ has been associated with formation of hypoacetylated repressive chromatin at the p21 promoter region in human breast cancer cells [139]. Increased level of reactive oxygen species (ROS) resulting in oxidative stress has been implicated in carcinogenesis by transcriptional regulation of selected genes which are involved in cell migration and motility, inter alia *MMPs, E-cadherin*, or *Snail*, a key transcription factor regulating epithelial-mesenchymal transition (EMT) [140]. It has been demonstrated that adaptive response to decreased levels of chronic oxidative stress induces malignant transformation of immortalized renal tubular epithelial cells, potentially throughout acquisition of EMT and stem cell characteristics [136]. 

## 7. Clinical Aspects of Global Histone Modification, including Acetylation and Methylation 

It has been investigated that global histone modifications are associated with various types of cancer. In some types of cancers the cellular expression of global histone acetylation or methylation is linked with patients’ outcomes.

Epigenetic changes, including H3K9Ac, H3K18Ac, and H4K16Ac, respectively, as well as H3K4Me2, H4K20Me3, and H4R3Meare, are considered as being potential biomarkers in patients with glioma. Relatively high median expression of H3K9Ac, H3K18Ac, and H4K16Ac (74%, 78%, and 68%, respectively), and H3K4Me2, H4K20Me3, and H4R3Me (63%, 75%, and 60%, respectively) has been detected in gliomas [56]. Interestingly, it has been shown that lower expression of H3K18Ac is connected with better survival among patients with glioblastoma, in contrast to patients with lung and kidney cancer, with whom lower levels of H3K18Ac and H3K4Me2 expression are associated with shorter survival [56,57]. Expression of seven biomarkers, including H3K9Ac, H3K18Ac, H4K12Ac, H4K16Ac, H4K20Me3, H3K4Me2, and H4R3Me2 have been detected in 880 breast carcinomas. Lower levels of histone modifications have been connected with unfavorable outcomes of patients, and higher levels of histone mark expression, excluding H4K20Me3, have been connected with better breast cancer-specific survival. Higher expressions of H3K9Ac, H3K18Ac, and H4R3Me2 have been correlated with longer disease-free survival and metastatic-specific survival (except for H4K20Me3 expression). In multivariate analysis only, H3K18Ac was an independent prognostic factor with respect to BCSS and DFS. Moreover, higher cellular levels of H3K9Ac, H3K18Ac, and H4K16Ac were present in tissues from patients who were not subjected to postoperative adjuvant treatment and who were connected with longer BCSS, DFS, and metastatic-specific survival. On the contrary, the correlations between patients’ outcomes and the level of histone marks diminished in patients treated with hormonal therapy [57].

Another study has discovered lower levels of H3K18Ac and H4K20Me3 in malignant breast tumors (MBT) compared to benign breast tumors (BBT). Additionally, H3K9ac levels were negatively correlated with the size of malignant tumors. These findings support the view that deacetylation of histones drives tumorigenic process [55].

In gastric carcinoma, trimethylation of H3K9 has been positively correlated with tumor stage, cancer recurrence, lymphovascular invasion, and independently predicted poor survival. Higher cellular levels of H3K9Ac have been correlated with poorly differentiated adenocarcinomas and loss of H3K9Ac expression has been associated with intestinal-type tumors [63]. Another study showed that the levels of H3K9Ac and H4K16Ac are increased at the promoter region of the bone morphogenetic 8B (BMP8B) gene in gastric cancer. Interestingly, these modifications were found not only in gastric cancer but in non-tumor tissues as well. The reduction of H3K9Ac and H4K16Ac expressions at the BMP8B promoter region has been connected with poorly-differentiated gastric cancer in comparison with moderately differentiated tumors. Moreover, decreased H3K9Ac at the BMP8B promoter region has been associated with histological diffuse-type gastric cancer compared to the intestinal-type of this neoplasm [141].

It has been discovered that H3K18Ac, H3K4Me2, and H3K9Me2 may serve as biomarkers in pancreatic adenocarcinoma. Low cellular levels of H3K18Ac, H3K4Me2, or H3K9Me2 are independent predictors of poor survival. Moreover, the combined low levels of H3K18Ac and/or H3K4Me2 have been found to be the best predictor of overall survival [66]. Another study has confirmed that high levels of H3K18Ac and H4K12Ac are independently associated with poor survival of patients with pancreatic cancer. The expression of H3K9Ac, H3K18Ac, and H4K12Ac was found to be low in normal areas adjacent to the tumor, and metastatic lesions had higher expression of histone acetylation compared to normal and primary pancreatic cancers. H3K12Ac expression was correlated to the grade of tumor, and global acetylation of all analyzed histones was associated with tumor stage [109]. Another study analyzed the correlation between H3K9Ac, H3K18Ac, H3K4Me2, H3K4Me3, and H3K9Me2 expression and outcomes for patients with pancreatic cancer treated with postoperative gemcitabine chemotherapy. Low H3K9Me2 levels have been detected in poorly differentiated adenocarcinomas or in other histological types than adenocarcinomas. High expression of H3K4Me3 has been found in well and moderately differentiated cancers. There are no differences in OR and DFS according to the expression level of any histone modification; however, a low level of H3K4Me2 expression has been independently associated with shorter DFS in the group of patients that received chemotherapy with the full-dose of gemcitabine [142].

Analysis of global histone modifications (H2AK5Ac, H3K9Ac, H4K8Ac, H2BK12Ac, and H3K4Me2) in non-small-cell lung cancer (NSCLC) tissues has revealed that patients with pathologic tumor stage II and low expression of H2AK5Ac have worse survival. Furthermore, high H3K4Me2 expression is connected with a better survival of patients with stage I large-cell carcinomas (LCC) or squamous cell carcinomas (SCC). Additionally, lower H3K9Ac expression is associated with better survival for patients with stage I adenocarcinomas [143]. Another study has shown that low expression of H4K5Ac and H4K8Ac, and high expression of H4K12Ac and H4K16Ac, are present in parenchyma of normal lungs compared to cancer cells. Higher H4K5Ac and H4K8Ac levels have been observed in squamous cell carcinoma, whereas low expression of H4K12Ac has been found in adenocarcinoma. A loss of trimethylation of H4K20 has been observed in lung tumors compared with normal lungs, and the H4K20Me3 expression was found to decrease with disease progression from cell hyperplasia to metaplasia, dysplasia, and then to carcinoma in situ. Patients with stage 1 adenocarcinomas expressing higher levels of H4K20Me3 had longer cancer-related survival. Moreover, decreased levels of H4K20Me3 have been connected with lower expression of specific H4K20 methyltransferase (SUV420H2), which is involved in the maintenance of telomere length [144]. The SUV420H2 is considered a promoter of EMT in pancreatic cancer cells, and this histone methyltransferase inhibits expression of transcription factors, including OVOL1, OVOL2, and FOXA1, which are indispensable for MET promotion through their repressive mark trimethylated H4K20 [145].

Global levels of histone 4 and histone 3 acetylation could be considered a biomarker in esophageal squamous cell carcinoma. Decreased global acetylation of both histones has been discovered in ESCC tissues in comparison to healthy esophageal tissues. In contrast, enhanced methylation of H3K4 and H3K27 has been detected in ESCC tissues in comparison to a control. Moreover, tumor TNM (Classification of Malignant Tumors) staging has been associated with hypoacetylation of H3 and hypermethylation of H3K27, whereas tumor histological differentiation has been correlated with H3 hypoacetylation and H3K4 and H3K27 hypermethylation. Furthermore, hypoacetylation of H3 has been associated with lymphatic permeation and hypoacetylation of H4 has been correlated with a history of alcohol consumption. Additionally, it has been discovered that mRNA levels of SIRT1, HDAC1, HDAC2, and SUV39H1 are higher in tumor tissue compared with a control tissue, and increase in advanced (stage 3 and 4) compared to earlier (stage 1 and 2) ESCCs. These findings suggest that overexpression of these genes is connected with the severity of the disease [61].

In hepatocellular cancer (HCC), high expression of H3K9Me2 has been detected in 64.8% of tumors. Moreover, H3K9Me2 has been found to be positively correlated with histone lysine methyltransferase G9a in tumor tissues, which indicates that interference of G9a may become a novel epigenetic treatment of HCC [146].

In colorectal cancer (CRC), expression levels of HDAC7 are increased and have been seen to be connected with H3K9, H3K18, and H4 hypoacetylation at the promoter regions for concentrative nucleoside transporter 2 (CNT2). This hypoacetylation leads to the compression of chromatin structure and reduction of CNT2 expression, which suggests that histone hypoacetylation via HDAC7 is responsible for CNT2 repression in CRC. Importantly, inhibition of histone deacetylase in HCT15 and HT29 human colon cancer cell lines increases cell uptake of nucleoside anti-cancer agent cladribine and decreases its IC50. These results indicate that combining nucleoside anti-cancer agents with inhibitors of histone deacetylases can overcome drug resistance in CRC cells [60]. In another study it was discovered that H3K9Ac, H3K4Me3, and H3K9Me3 expression is regularly observed in CRC (75% of tumor samples, and 100% and 77%, respectively), and exclusive acetylation of H3K9 and trimethylation of H3K4 are present in 13% of tumor samples. Lower levels of repressive histone mark (H3K9Me3) have been detected in higher stages of tumors, but there have been no correlations between H3K9Ac, H3K4Me3, and HDAC1-3 [58]. Interestingly, global H3K9Me2 expression has been seen to be higher in adenoma and colorectal cancer cells compared to normal glandular cells [59].

In oral cancer, H3K9 hypoacetylation is contemplated as a marker of poor prognosis. The level of H3K9Ac expression is lower in oral squamous cell carcinoma (OSCC) compared with oral leukoplakia (OL), the most common precursor of OSCC. In survival analysis lower expression of H3K9Ac in OSCC is associated with a poorer outcome [68].

H3K27Me3 is considered a biomarker in malignant peripheral nerve sheath tumors (MNSTs). The expression of H3K27Me3 has been found to be lost in 54% of MNSTs, but it has been retained in non-MPNSTs tumors [147]. H3K27Me3 has been observed to be present in 56% of malignant peripheral nerve sheath tumors (MPNST), 91% of synovial sarcomas (SS), and 90% of fibrosarcomatous dermatofibrosarcoma protuberans (FDP). H3K27Me3 expression in MPNST is not correlated with OS or PFS [148].

The expression of H3K27Me3 is also present in diffuse large B-cell lymphoma (DLBCL) cells, and high levels of H3K27 trimethylation are an independent prognostic marker for lower OS [54].

Analysis of global expression of H3Ac and H4Ac, and H3K18Ac in urothelial bladder cancer (BCA), including non-muscle invasive bladder cancer (NMIBC) and muscle invasive bladder cancer (MIBC), as well as in normal urothelial tissue, has revealed that global H3Ac expression is lower in BCA than in normal tissue, but global expression of H4Ac and H3K18Ac is similar. Histone acetylation decreases during progression of NMICB to MIBC, but histone acetylation is not associated with PFS or cancer-specific survival in patients with NMIBC or MIBC [55].

Taken together, these results indicate that aberrations of global histone modifications, including acetylation and methylation, influence growth and progression of a wide range of tumors. Additionally, histone modification patterns are useful as biomarkers of clinical outcomes. Further research regarding histone modifications may contribute to the development of new cancer treatment strategies.

The histone marks and their associations with OS and DFS are presented in Table 2.

## 8. Potential Strategies for Pharmacological Targeting H3K18

DCH36_06, a potent inhibitor of p300/CBP, decreases acetylation of H3K18 in leukemic cells, and as a result inhibits cell proliferation, arrests cell cycles, and induces apoptosis by activation of capase 3, caspase 9, and PARP [149].

The I-CBP112, a ligand for p300/CBP bromodomains, stimulates the acetylation of H3K18 in acute leukemia and prostate cancer cells and exerts antiproliferative effects that reveal a novel strategy for treating cancer by modulating histone acetylation [150].

Two alkylating agents, bendamustine and a metabolite of cyclophosphamide (4-hydroperoxy-cyclophosphamide, 4-OHCY) induce H3K18 acetylation and decrease the expression of SIRT1, SIRT7, and HDAC3 in a dose-dependent manner in mantle cell lymphoma (MCL) cells [151]. Furthermore, the combination of bendamustine or 4-OHCY with HDAC1 and HDAC2 inhibitors results in synergistic effects on histone acetylation and cytotoxicity. Discovery of the novel function of alkylating agents that target cancer-specific histone modification (H3K18 hypoacetylation) provides a rationale for new anti-cancer treatment regimens involving HDAC inhibitors [151].

## 9. Future Perspectives on Epigenetic Anti-Cancer Therapy

Currently, epigenetic therapy is one of the most dynamically developing areas of preclinical and clinical studies. It involves the developing of drugs or techniques targeting chromatin remodeling mechanisms that participate in carcinogenesis [151,152]. The benefits of epigenetic therapies may be limited by their side effects compared to standard anticancer treatment. Nowadays, different classes of drugs are being invented and then investigated for their usefulness as novel anti-cancer agents. One such class of drugs, comprising HDAC inhibitors (HDIs), have either been approved by the FDA or are in clinical trials. The most common HDIs are presented below.

Vorinostat has been approved for the treatment of cutaneous T-cell lymphoma (CTCL) and is in clinical trials for treatment of multiple myeloma, mesothelioma, neuroblastoma, and glioblastoma. Romidepsin has been approved in CTCL and periphelial T-cell lymphoma and is in clinical trials for the treatment of multiple myeloma, breast cancer, sarcoma, SCLC and lymphoma. Belinostat has been approved for treatment of multiple malynoma and is in clinical trials for the treatment of hepatocarcinoma, NSCLC, ovarian cancer, soft tissue sarcoma, and prostate cancer. Panobinostat has been approved in CTCL and is in clinical trials for treatment of multiple malynoma, metastatic melanoma, and prostate cancer [37].

The limitations of HDIs are toxic effects, resistance of cancer cells, and limited usefulness in treating solid tumors. Additionally, there is a need to discover biomarkers to either select or predict a response of patients to HDIs [152]. The evaluation of histone acetylation levels in cancer cells may help to estimate the range of response to HDIs. In particular, the investigation of cellular levels of H3K18 modifications may reveal innovative methods in anti-cancer therapy. As H3K18Ac is a selectively deacetylated by SIRT7 [153], SIRT7 may be considered a promising drug target. The development of SIRT7 modulators (inhibitors and activators) may become crucial for developing innovative therapeutic strategies based on chromatin reprogramming. A few organic compounds, which could be potential selective SIRT7 inhibitors, have been discovered [154,155]. However, knowledge about SIRT7 function still needs to be expanded.

## 10. Conclusions

Histone acetylation is one of the most common histone epigenetic modifications that regulates DNA transcriptional machinery and in turn impacts gene expression. Opposing the reaction to acetylation is deacetylation. The balance between these reactions is indispensable for assuring chromatin integrity. Histone acetylation occurs in cancer cells, and it may play a dual role in cancer progression. It may participate in the silencing of tumor-suppressor genes and, on the other hand, it is able to enhance expression of oncogenes.

Cancer is a consequence of aberrations in the regulation of these processes as inappropriate activation of oncogenes and inactivation of tumor suppressors results in neoplastic transformation [156]. At present, cancer therapy becomes more personalized and specifically thanks to the implementation of biomarkers, it allows for better identification of the appropriate therapy for patients. New strategies based on the identification and direct evaluation of activity of histone modifiers, as well as cellular levels of histone modifications, are highly needed in order to predict prognosis and adjust chemotherapy.

Cellular levels of histone acetylation are considered to be a biomarker of cancer progression and are a potential target of an anti-cancer therapy. Expression of histone acetylation has been investigated in vitro as well as in in vivo settings. In vitro studies have demonstrated that acetylation affects many pathways involved in carcinogenesis in a wide range of different cancer cells.

Clinical research has revealed that the expression of histone acetylation in cancer tissues is often correlated with tumor differentiation and its size and disease stage. The aberrant level of global histone acetylation is usually associated with outcomes, including OS and DFS, in patients with different types of cancer.

One of the most common local histone modifications is acetylation of histone 3 at position K18 (H3K18Ac). Aberrant H3K18Ac levels have been found in blood, brain, breast, colon, esophageal, gastric, liver, lung, pancreas, prostate, and oral cancers. In many cancers low H3K18Ac levels are correlated with poor patient outcomes. However, in some neoplasms, including hepatocellular carcinoma and glioblastoma, low H3K18Ac levels are associated with better patient prognosis, which indicates that H3K18Ac is able to predict prognosis for patients with different types of cancer, and that the histone marks can provide tissue-specific features.

H3K18Ac is an important prognostic factor in patients with different types of cancer, although its detailed participation in carcinogenesis requires further investigation.

Taken together, screening for histone modifications in cancer tissues helps to better understand molecular events that occur during carcinogenesis and it is useful as prognostic tool.

## Figures and Tables

**Table 1:** Expression of histone acetylation and methylation markers in cancer cell lines.

Cell Origin	Experimental Model	Marker Expression	References
**Brain**	BV2 microglia cells C6 glioma cells GL261 glioma cells in vitro	H4K16Ac ↑ in BV2 microglia upon coculture with C6 glioma cells, GL261 glioma cells, or murine primary glioma tumorspheres	[102]
	U87glioblastoma cells in vitro	H3K56Ac ↓ on the promoters of glycolytic genes after knockdown of *mTORC2*	[101]
**Breast**	MCF10A (non-cancerous cell line with characteristics of epithelial hyperplasia) MCF7 (mature luminal subtype of breast cancer cell line expressing estrogen and progesterone receptors) MDA-MB-231 (triple negative (ESR1-, PGR-, HER2-) invasive metastatic adenocarcinoma cell line) in vitro	Global H3K4Ac ↑ in cancer cell lines (MCF7, MDA-MB-231) and H3K4Me3 in MDA-MB-231 metastatic cell line compared to a normal breast epithelial cell line (MCF10A)	[93]
	MDA-MB-231 (triple negative) MCF-7 (ER+) with transfected TIP60 grafted to athymic Balb-c mice in vivo	H3K4Ac ↑ (in cells with TIP60) H3K4Ac ↓ (in cells without TIP60)	[8]
**Cervix**	HeLa cervical carcinoma cells	H3K56Ac ↑ after EGF treatment	[101]
**Esophagus**	ESCC cell lines Eca-109 and TE-1 in vitro	H3K27Ac ↑ at the promoter of CCAT1 in ESCC cells (Eca-109) compared with normal human esophageal epithelial cells (HET-1A)	[97]
**Oral cavity**	OSCC cell lines SCC-9 and CAL-27 in vitro Male nude mice in vivo	H3K27Ac ↑ at the promoter of PLAC2 in OSCC cells and normal epithelial HOK cells	[98]
**Prostate**	PC3 prostate cancer cells PC3/Doc docetaxel-resistant PC3 cells in vitro	↑ acetylated H3 and H4 in resistant cells compared to PC3 cells. TSA, an HDAC inhibitor, ↑ acetylation of histones H3K9, H4K8, and H4K16	[103]

H, histone; K, lysine; Ac, acetylated; mTORC2, Mammalian target of rapamycin complex 2; EGF, epidermal growth factor; ESCC, esophageal squamous cell carcinoma; CCAT1, colon cancer associated transcript-1; OSCC, oral squamous cell carcinoma; PLAC2, placenta-specific protein 2; TSA, Trichostatin A; ↑ increased level, ↓ decreased level.

**Table 2:** Global histone acetylation and methylation in relation to survival of patients with cancer.

Tumor Localization	Type of Cancer	Assay	Marker	Expression in Tumor Cells	Patients Survival	Reference
Bladder	271 urothelial bladder cancers	TMA	H3Ac H4Ac H3K18Ac	Lower H3Ac levels in cancer than in normal urothelial tissue but similar levels of H4Ac and H3K18Ac	No correlation of histone acetylation and PFS or CSS	[55]
Blood	231 diffuse large B-cell lymphomas (DLBCLs)	TMA	H3K27Me3	High expression in 35.7% of cases	Lower survival for patients with high expression of H3K27Me3 (independent predictor)	[54]
Brain	230 gliomas (WHO grades 1–4)	IHC	H3K9Ac	Broad distribution of staining (mean 70% of tumor cells)	Lower survival for patients with grade 1–2 astrocytomas with H3K9Ac in less than 88% of tumor cells	[56]
Breast	880 breast cancers of different histology	TMA	H3K9Ac H3K18Ac H4K12Ac H4K16Ac H3K4Me2 H4K20Me3	Low score for H4K16Ac in 78.9% of, and high scores for H3K18Ac and H4K20Me3 in 81.4% and 69.8% of breast tumors, respectively. About 50%/50% of low/high scores for other markers (H3K9Ac, H4K12Ac, H4R3Me2, and H3K4Me2)	Lower BCSS and metastatic-specific survival for patients with low (<100) marker scores (except H4K20Me3). Lower DFS for patients with low scores for H3K18Ac, H4R3Me2, and H3K9Ac.	[57]
Deep soft tissues	131 sarcomas including 82 SS, 39 MPNST, and 10 FDP	IHC and TMA	H3K27Me3	Broad distribution of staining	No association between H3K27Me3 expression in MPNST and PFS or OS	[148]
Lung	285 lung cancers (stages 1–4)	TMA	H3K18Ac H3K4Me2	Broad distribution of staining for both H3K18Ac and H3K4Me2	Lower 15-year survival for patients with stage 1 lung adenocarcinomas with lower expression of both modifications (independent predictor)	[57]
	138 early-stage NSCLCs (stage I to IIIA)	IHC	H2AK5Ac	Broad distribution of staining (mean 68% of tumor cells)	Lower survival for patients with stage II NSCLCs with H2AK5Ac in less than 5% of tumor cells	[143]
		IHC	H3K9Ac	Broad distribution of staining (mean 42% of tumor cells)	Lower survival for patients with stage I adenocarcinomas with H3K9Ac in more than 68% of tumor cells	
		IHC	H3K4Me2	Broad distribution of staining (mean 57% of tumor cells)	Lower survival for patients with stage I large-cell or squamous cell carcinomas with H3K4Me2 in less than 85% of tumor cells	
	157 stage I–IV lung cancers (50 squamous cell carcinomas and 107 adenocarcinomas)	IHC	H4K20Me3	H4K20Me3 score between 0 and 100 in 70% of the tumor samples	Lower survival for patients with stage I adenocarcinomas with a low (≤100) H4K20Me3 score	[144]
Pancreas	229 pancreatic adenocarcinomas	TMA	H3K18Ac H3K4Me2 H3K9Me2	Broad distribution of staining for each modification	Low levels of H3K4Me2, H3K9Me2, and H3K18Ac (<60%, <25%, and <35% of tumor cells, respectively) were each independent predictors of lower OS for patients with stages I and II of the disease. Lower OS for patients treated with postoperative chemoradiotherapy with low H3K4Me2 or H3K9Me2 staining (each was an independent predictor)	[66]
	119 pancreatic cancers (stage I–IV)		H3K9Ac H3K18Ac H4K12Ac	Median percentage of stained cells for H3K9Ac (80%), H3K18Ac (65%), and H4K12Ac (60%)	High expression (H score ≥100) of H3K18Ac and H4K12Ac were both independently associated with shorter OS	[109]
	61 pancreatic cancers (stage IB–III)	TMA	H3K9Ac H3K18Ac H3K4Me2 H3K4Me3 H3K9Me2	The median H-scores were: H3K9me2, 158; H3K9ac, 140; H3K4me2, 142; H3K4me3, 160; H3K18ac, 162.	None of histone modifications influenced OS or DFS.	[109]
Stomach	261 gastric carcinomas (stage I–IV)	TMA	H3K9Ac H4K16Ac H4K20Me3	Strong staining (score = 4) for H3K6Ac, H4K16Ac, and H4K20Me3 in most cases (49%, 60.9%, and 54.4%, respectively). Strong staining for H3K9Me3 in only 33% of the cases.	No influence of H3K9Ac, H4K16Ac, or H4K20Me3 expression on survival. Lower survival for patients with strong H3K9Me3 staining (independent predictor).	[63]
Oral	33 OL and 86 OSCC	IHC	H3K9Ac	Staining in 81% NOM cells, 94.4% OL cells, and 78.2% OSCC cells	Lower survival for patients with low expression	[68]

Legend: WHO, World Health Organization; TMA, tissue microarrays; PFS, progression-free survival; CSS, cancer-specific survival; IHC, immunohistochemistry; BCSS, breast cancer-specific survival; DFS, disease-free survival; OS, overall survival; SS, synovial sarcoma; MPNST, malignant peripheral nerve sheath tumor; FDP, fibrosarcomatous dermatofibrosarcoma protuberans; NSCLC, non-small-cell lung cancer; OL, oral leucoplakia; OSCC, oral squamous cell carcinoma; NOM, normal oral mucosa; DLBCLs, diffuse large B-cell lymphomas.
